# Monocytosis in the acute phase of SARS-CoV-2 infection predicts the presence of anosognosia for cognitive deficits in the chronic phase

**DOI:** 10.1016/j.bbih.2022.100511

**Published:** 2022-09-16

**Authors:** A. Nuber-Champier, P. Voruz, I. Jacot de Alcântara, G. Breville, G. Allali, P.H. Lalive, F. Assal, J.A. Péron

**Affiliations:** a Clinical and Experimental Neuropsychology Laboratory, Faculty of Psychology, University of Geneva, Switzerland; b Neurology Division, Geneva University Hospitals, Switzerland; c Faculty of Medicine, University of Geneva, Switzerland; d Leenaards Memory Center, Lausanne University Hospital and University of Lausanne, Switzerland

**Keywords:** Anosognosia, Immunology, Monocytes, Cognition, Neuropsychology, SARS-CoV-2, Post-COVID-19 condition, (HIV), human immunodeficiency virus, (HCoV), human coronavirus, (HUG), Geneva University Hospitals, (ICU), intensive care unit, (ROC), receiver operating characteristic, (CRP), C-reactive protein, (SAE), sepsis-associated encephalopathy, (RT-PCR), reverse transcription polymerase chain reaction, (FDR), false discovery rate, (AUC), Area under the curve

## Abstract

Reduced awareness of neuropsychological disorders (i.e., anosognosia) is a striking symptom of post-COVID-19 condition. Some leukocyte markers in the acute phase may predict the presence of anosognosia in the chronic phase, but they have not yet been identified. This study aimed to determine whether patients with anosognosia for their memory deficits in the chronic phase presented specific leukocyte distribution in the acute phase, and if so, whether these leukocyte levels might be predictive of anosognosia.

First, we compared the acute immunological data (i.e., white blood cell differentiation count) of 20 patients who displayed anosognosia 6–9 months after being infected with SARS-CoV-2 (230.25 ± 46.65 days) versus 41 patients infected with SARS-Cov-2 who did not develop anosognosia. Second, we performed an ROC analysis to evaluate the predictive value of the leukocyte markers that emerged from this comparison.

Blood circulating monocytes (%) in the acute phase of SARS-CoV-2 infection were associated with long-term post-COVID-19 anosognosia. A monocyte percentage of 7.35% of the total number of leukocytes at admission seemed to predict the presence of chronic anosognosia 6–9 months after infection.

## 1 Introduction

The persistence of cognitive symptoms following SARS-CoV-2 infection is reflected in the presence of memory deficits and impaired executive, instrumental and attentional functioning several weeks and even months post-discharge (Alemanno et al., 2021; Almeria et al., 2020; Voruz et al., 2022a). These impairments can be severe, even in individuals with no previous neurological conditions (Almeria et al., 2020). Initial observations point to fairly heterogeneous neuropsychological profiles, one striking clinical feature being the presence of impaired awareness of neuropsychological deficits, referred to as *anosognosia* (Voruz et al., 2022a; Parsons et al., 2021). Some patients complain of extremely severe cognitive problems but have no objective disorders, whereas others have no subjective complaints but exhibit severe cognitive disorders (Almeria et al., 2020; Voruz et al., 2022a). In this regard, interesting information can be derived from other types of human coronavirus (HCoV). In the past, neuroinvasive types of HCoV have reportedly induced both acute and chronic complications for cognition and consciousness (Desforges et al., 2014). HCoV-OC43 may even contribute to the development of neurodegenerative cascades (Meessen-Pinard et al., 2017), with brain regions injured by HCoV viral attack (Netland et al., 2008) overlapping with those responsible for self-consciousness, as observed in SARS-CoV-2 (Parsons et al., 2021). Interestingly, in patients with human immunodeficiency virus (HIV) (Cysique et al., 2012; Casaletto et al., 2014), Alzheimer's disease (Starkstein, 2014) or multiple sclerosis (Reich et al., 2015), anosognosia (Starkstein et al., 2006) is predictive of the presence and intensity of neuropsychological symptoms: the more anosognosic the patients, the more severe their neuropsychological deficits (Starkstein, 2014). SARS-CoV-2 has been observed to cause cognitive and neurological damage in a similar way to these pathologies. Voruz et al. (2022a) recently demonstrated that patients who are anosognosic 6–9 months after SARS-CoV-2 infection have significantly more severe memory deficits than nosognosic patients. They also have fewer self-reported psychiatric symptoms and a better self-reported quality of life. These post-COVID-19 cognitive findings in anosognosic patients are mainly supported by hypoconnectivity between frontal and dorsolateral prefrontal regions, somatosensory networks, and Lobules I-IV and V of the cerebellum (Voruz et al., 2022a). This post-COVID-19 neurocognitive phenotype therefore seems to be congruent with the neurodegenerative, viral, and autoimmune pathologies mentioned previously.

Another striking observation regarding post-COVID-19 condition is that the severity of the respiratory form in the acute phase does not seem to be the best predictor of cognitive impairment in the chronic phase (Voruz et al., 2022a; Crook et al., 2021). In a recent literature review, intrinsic risk factors such as genetics, lifestyle, and immunological profile were better predictors of the pathophysiological consequences of SARS-CoV-2 infection (e.g., neuroinflammation, cytokine cascade, hypercoagulability, direct brain injury, astrocyte infection) that ultimately lead to the development of cognitive impairment (Damiano et al., 2021). In this context, we wondered whether acute-phase biological markers other than respiratory distress might predict the presence of cognitive deficits, particularly anosognosia, which is known to be correlated with severe neuropsychological syndromes (Voruz et al., 2022a; Starkstein, 2014). Identifying these predictors might allow for the development of individualized and targeted management for patients most likely to exhibit severe cognitive impairments. This is, nevertheless, no easy task, as the pathways associated with the neurotropism of SARS-CoV-2 are not yet well established (for reviews, see (Crook et al., 2021; Damiano et al., 2021).

To investigate the neurotropism of SARS-CoV-2, we focused here on the hypothesis of an indirect effect of leukocyte variation on cognition (Bougakov et al., 2021; Íde Figueiredo et al., 2020). Patterns of dysregulation of the complex immune system during COVID-19 have been associated with the severity and final outcome of the infection (Giamarellos-Bourboulis et al., 2020; Lombardi et al., 2020). One review suggested that monocyte-derived macrophages are a characteristic target of SARS-CoV-2 (Meidaninikjeh et al., 2021), while studies have pointed to the persistence of pro-inflammatory immune dysregulation after infection (Damiano et al., 2021; Meidaninikjeh et al., 2021; Mazza et al., 2021). Interestingly, these phenomena seem to be linked to cognitive deficits and psychiatric symptoms up to 3 months post-infection (Mazza et al., 2021; Zhou et al., 2020), but results remain limited, and no study has so far comprehensively assessed the association between overall cognitive function and acute immunological profiles. Associations between immunological aspects and cognitive deficits have been observed in several neurocognitive pathologies following an infection, including HIV (Kusao et al., 2012) and sepsis-associated encephalopathy (SAE) (Andonegui et al., 2018; Chung et al., 2020; Michels et al., 2015; Trzeciak et al., 2020), as well as in neurodegenerative diseases (Baufeld et al., 2018). Taken together, these observations suggest that the interaction between dysregulation of the immune system in the acute phase of SARS-CoV-2 infection and long-term cognitive deficits, particularly anosognosia, is an interesting lead to follow.

In the present study, therefore, we further investigated the immune-related etiology of neurocognitive post-COVID-19 condition. Our objective was to determine whether patients who go on to exhibit anosognosia in the chronic phase differ significantly on white blood cell differential count in the acute phase from those who do not, and whether any such significant differences are predictive of this chronic lack of awareness. In line with the results of Cervia et al*.* (Cervia et al., 2022/01) showing that the immunoglobulin signature predicts the risk of post-COVID-19 condition, and based on the hypothesis of immune damage to the central nervous system in COVID-19 (Voruz et al., 2022a; Meidaninikjeh et al., 2021) and other viral or neurodegenerative diseases (e.g., HIV, Alzheimer's disease) (Muñoz-Nevárez et al., 2020; Garré and Yang, 2018),we hypothesized that innate immunity biomarkers measured in the acute phase of SARS-CoV-2 infection can be used to distinguish anosognosic from nosognosic patients 6–9 months post-infection (chronic phase). More specifically, based on research showing that anosognosic patients with Alzheimer's disease or post-COVID-19 condition (Voruz et al., 2022a) have more marked cognitive deficits than nosognosic patients (Starkstein, 2014), with elevated inflammatory markers (Holmes et al., 2009), we hypothesized that anosognosic patients with post–COVID-19 condition had higher levels of various leukocyte biomarkers than nosognosic patients in the acute phase. Finally, we hypothesized that these markers are predictive of anosognosia 6–9 months after COVID-19.

## 2 Method

### 2.1 Participants (Table 1)

The sample comprised 61 patients with SARS-CoV-2 infection drawn from the COVID-COG cohort of Geneva University Hospitals (HUG) (Voruz et al., 2022), who were assessed 6–9 months after being admitted to hospital. We selected patients with no previous history of cognitive deficits or neuropsychiatric disease. Blood samples were collected from March 16, 2020 to February 8, 2021, and none of the patients received anti-SARS-CoV-2 mAbs. Within the sample, 38 had had moderate symptoms (conventional hospitalization without mechanical ventilation) in the acute phase, and 23 had had severe symptoms requiring a stay in intensive care unit (ICU) and mechanical ventilation. SARS-CoV-2 infection was detected using a reverse transcription polymerase chain reaction (RT-PCR) test. This technique allows the N and E genes to be detected with the LightCycler 480 system (Roche, Switzerland). In rare cases, where PCR testing was not available for clinical reasons, intrathecal IgG synthesis was used for diagnostic purposes and to confirm SARS-CoV-2 infection. As part of the COVID-COG study, all patients completed a battery of neurological, neuropsychological and psychiatric tests and questionnaires 230.25 ± 46.65 days following SARS-CoV-2 infection. For the present study, only sociodemographic data, clinical history, objective memory tests, and self-reported cognitive complaints related to memory disorders were extracted.

#### 2.1.1 Subdivision of patients according to their anosognosia score

Patients were divided into two groups according to their anosognosia for memory disturbances, measured 6–9 months after SARS-CoV-2 infection: 1) anosognosic for memory dysfunctions (*n* = 20), versus 2) nosognosic for memory functions/dysfunctions (*n* = 41). This was done independently of the severity of their respiratory symptoms in the acute phase of the disease (anosognosic: *n* = 12 moderate and *n* = 8 severe vs. nosognosic: *n* = 26 moderate and *n* = 15 severe). Anosognosia was measured as follows: scores on the self-report Cognitive Complaints Questionnaire (Thomas-Anterion et al., 2004) were first standardized and divided into four categories: 0 = normal behavior, 1 = limited influence on daily life, 2 = noticeable influence on daily life, and 3 = substantial influence on daily life. Each standardized score on this subjective measure was then subtracted from the standardized scores on objective measures of memory. Short-term memory was assessed with forward digit spans (Drozdick et al., 2018) and the Corsi test (Kessels et al., 2000/12), and episodic memory with the 16-item free/cued recall paradigm (Van der Linden et al., 2004) and the delayed recall of the Rey-Osterrieth Complex Figure test (Meyers and Meyers, 1995). The resulting self-appraisal discrepancy scores could therefore range from −3 to 3, with any score below 0 indicating anosognosia. For example, patients who reported no memory disorders (Cognitive Complaints Questionnaire score = 3), but performed very poorly on verbal episodic memory (delayed free recall test score = 0), were deemed to exhibit anosognosia for memory dysfunction (0 [standardized score on episodic memory tests] - 3 [score on self-report Cognitive Complaints Questionnaire] = −3). The method for calculating anosognosia described in this study was initially validated in Tondelli et al. (2018), and subsequently used by Voruz et al., 2022a, 2022b. Of note, the Behavior Rating Inventory of Executive Function - Adult Version (Roth et al., 2005) was used to measure the validity of the patients' responses, as well as the presence of any noncredible symptoms. In addition to this calculation of anosognosia by self-report discrepancy scores, clinical observation of anosognosia was performed during the neuropsychological clinical assessment by board-certified psychologists (P.V., A.N.-C., I.J.-A.).

#### 2.1.2 Power analysis

We performed a power analysis of the number of participants required, using the following equation: $N\;=\;\frac{2\;\times \;\sigma^{2}\;\left(z_{\frac{\alpha }{2}}+z_{\beta }\right)\;}{{\left({\bar{\text{X}}}_{1}-\;{\bar{\text{X}}}_{2}\right)}^{2}}$. This calculation was based on previous studies that had examined the relationship between immunity and cognition in patients with HIV or SAE (Hopkins et al., 2004; Robertson et al., 2007). Relying on previous power analyses for HIV, 20 participants, with 10 per group (alpha = 0.05, beta = 0.2, and power = 0.8) was the total number of participants required for the study. As we intended to use nonparametric statistical tests, we added 15% more participants to the initial number needed (Lehmann and Rojo, 2012). We thus determined that the total number of participants required was 23 (i.e., 12 per group). Power calculations previously used for SAE yielded a total number of 34 persons. After adding the 15% owing to the use of nonparametric tests, we arrived at 39 persons (i.e., 19 persons per group; alpha = 0.05, beta = 0.2, and power = 0.8).

#### 2.1.3 Ethics

After being given a full description of the study, participants provided their written informed consent. The study was conducted in accordance with the Declaration of Helsinki, and the study protocol was approved by the cantonal ethics committee of Geneva (CER-02186).

### 2.2 Retrospective extraction of acute-phase physiological parameters

Immunological parameters were retrospectively extracted from HUG's internal database. We only selected physiological parameters measured on admission to hospital (1.35 ± 3.41 days after a positive RT-PCR test for anosognosic patients vs. 2.05 ± 3.50 days for nosognosic patients), to avoid any effect of subsequent medication and oxygen therapy. We also extracted hematological, metabolic and cardiac parameters, as we knew that these variables might have an impact on cognition, given that they can contribute significantly to the development of cognitive deficits in dementia (Eggermont et al., 2012). Parameters were measured by the diagnostic department of HUG's Gas Testing, Hematology and Virology Laboratory. All laboratory samples were analyzed with Piccolo Xpress (Sysmex, Switzerland) tools and an ABL blood gas analyzer (Radiometer RSCH GmbH, Switzerland) at HUG. The following leukocyte distribution parameters (venous blood) were extracted: percentage (%) and mass concentration (G/l) of lymphocytes, monocytes, basophils, eosinophils, and neutrophils. Only mass concentration (G/l) was extracted for leukocytes. Subsequently, based on previous studies in SARS-CoV-2 (Akinwumi et al., 2021), the following ratios were calculated: lymphocyte/monocyte ratio [lymphocyte (G/l) divided by monocyte (G/l)], lymphocyte/neutrophil ratio [lymphocyte (G/l) divided by neutrophil (G/l)], and neutrophil/monocyte ratio [neutrophil (G/l) divided by monocyte (G/l)]. Inflammation was also measured as C-reactive protein (CRP) (mg/l). Finally, we classified the patients according to the normal thresholds for each percentage of each immunological variable (we considered the normal range to be 33–80% for neutrophils, 0–5% for eosinophils, 0–2% for basophils, 0–9% for monocytes, and 15–60% for lymphocytes).

### 2.3 Other clinical variables (6–9 months post-infection)

#### 2.3.1 Sociodemographic and clinical data

In addition to age, collected during the inclusion interview, we recorded patients’ sex, handedness, and education level. To complement information about previous neurological, psychiatric, and developmental conditions and cancer collected during the inclusion interview, we asked patients about previous cardiovascular disease, respiratory disorders, immunosuppression status, sleep apnea syndrome, diabetes, and smoking. Participants were asked to describe the symptoms they had experienced, both during the acute phase of the infection and currently (6–9 months post-infection), and the number of days they had spent in hospital, where relevant.

### 2.4 Statistical analysis

Given the nonparametric distribution of our dataset, we performed intergroup (anosognosic vs. nosognosic) analyses on sociodemographic and immunological variables with nonparametric Mann-Whitney *U* tests for continuous data and chi-square tests for categorical variables, with a significance threshold of *p* = .05 false discovery rate (FDR) corrected. Moreover, we performed a receiver operating characteristic (ROC) analysis to identify the acute immunological variable(s) predictive of anosognosia 6–9 months after infection. For significant variables, we performed a Youden test to determine the best cut-off. We used a significance level of *p* < .05 FDR corrected. All analyses were performed with SPSS Statistics version 28.0.1.

## 3 Results

### 3.1 Symptom validity and presence of noncredible symptoms

The measurement of symptom validity, congruence, and presence of noncredible symptoms using the Behavior Rating Inventory of Executive Function yielded good to excellent results for all participants.

### 3.2 Sociodemographic and clinical variables as a function of anosognosia for memory disorders in the chronic phase

No significant differences were observed between the two groups on either sociodemographic characteristics (i.e., age, handedness, sex, sociocultural level) or other clinical variables (see Table 1), with the exception of chronic renal failure (χ^2^ = 4.24, *p* = .040).

**Table 1:** Sociodemographic and clinical measures of patients with COVID-19 divided into two groups according to presence/absence of anosognosia 6–9 months post-infection.

	Anosognosic patients *n* = 20	Nosognosic patients *n* = 41	*p* < .050
Mean age in years (±*SD*)	58.40 (±13.57)	58.10 (±10.17)	.604
Education level (1/2/3)	2/6/12	1/14/26	.446
Sex (F/M)	5/15	15/26	.333
Number of patients who required conventional hospitalization/ICU in the acute phase	12/8	26/15	.851
Mean days of hospitalization (±*SD*)	24.60 (±24.35)	22.00 (±26.82)	.963
Mean days between positive RT-PCR test and collection of immunological data (±*SD*)	1.35 (+3.41)	2.05 (+3.50)	
Diabetes (Yes/No)	4/16	5/36	.328
History of respiratory disorders (Yes/No)	2/18	7/34	.465
History of cardiovascular disorders (Yes/No)	6/14	6/35	.156
History of neurological disorders (Yes/No)	0/20	0/41	1
History of psychiatric disorders (Yes/No)	0/20	1/40	.309
History of cancer (Yes/No)	0/20	0/41	1
History of severe immunosuppression (Yes/No)	0/20	0/41	1
History of developmental disorders (Yes/No)	0/20	0/41	1
Chronic renal failure (Yes/No)	2/18	0/41	.040*
Sleep apnea syndrome (Yes/No)	1/19	9/32	.093

*Note.* Education level: 1 = compulsory schooling, 2 = post-compulsory schooling, and 3 = university degree or equivalent. ICU: intensive care unit; RT-PCR: reverse transcription polymerase chain reaction allowing RNA to be quantified to determine SARS-CoV-2 infection. The nosognosic/anosognosic groups were formed according to awareness (or lack) of awareness of memory impairment 6–9 months after SARS-CoV-2 infection.

### 3.3 Monocyte percentage to total leukocytes in acute phase as a function of anosognosia for chronic-phase memory impairment (6–9 months post-infection)

**Fig. 1:**
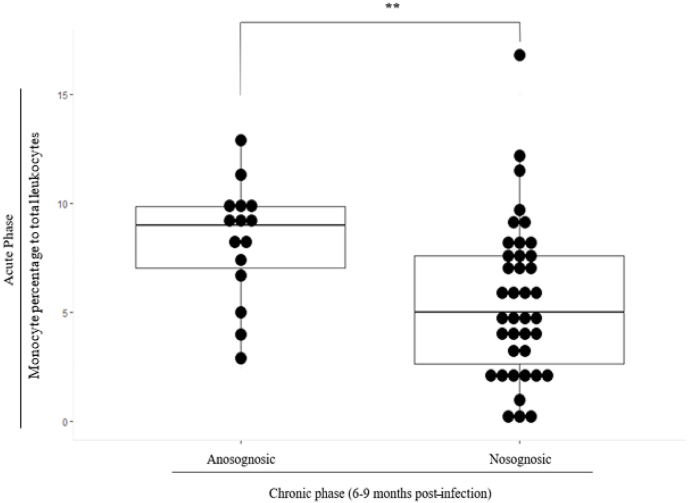
Higher mean monocyte percentages for anosognosic versus nosognosic patients (*p* < .05 FDR-corrected).

After FDR correction, the only surviving comparison concerned the monocyte percentage in the total number of leukocytes. Anosognosic patients had a significantly higher monocyte percentage than nosognosic patients (*z* = −2.87, *p* = .004, *r* = −0.387) (Fig. 1). Consistent with these results, more anosognosic patients had a monocyte percentage above the threshold defined as normal than nosognosic patients did (χ^2^ = 5.80, *p* = .016). The distribution of nosognosic and anosognosic patients according to the different immunological parameters is available in Supplementary Material (Table A1).

### 3.4 Immunological variables in acute phase as predictors of anosognosia 6–9 months post-SARS-CoV-2 infection

**Fig. 2:**
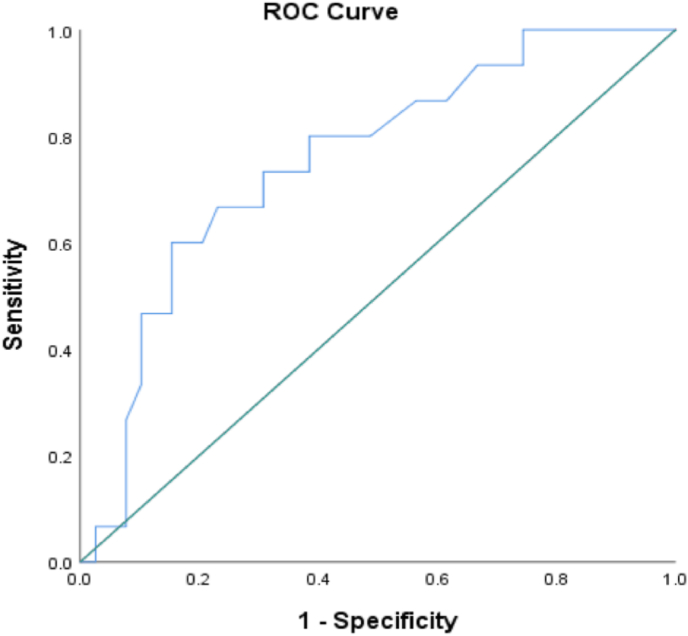
ROC curve analysis considering monocyte percentage of total leucocytes for prediction of anosognosia 6–9 months post-SARS-CoV-2 infection (AUC = 0.755, 95% CI [0.614, 0.895]) *Note.* The area under the curve (AUC) represents the propensity of the variable to predict anosognosia 6–9 months after infection. An area equivalent to 0.70 was considered a good predictor. We therefore observed that the monocyte percentage of total leukocytes predicted anosognosia 6–9 months after SARS-CoV-2 infection with an AUC of 0.755 and asymptotes in terms of sensitivity at 0.80 and specificity at .80.

An ROC curve analysis was performed on 52 of the 61 patients (anosognosic: *n* = 15; nosognosic: *n* = 37), as we analyzed one measure of each immunological parameter, and nine patients for whom we did not have all the immunological measures were therefore excluded. This analysis revealed that monocyte percentage in the total number of leukocytes (*p* = .004) in the acute phase significantly predicted anosognosia 6–9 months post-infection. For each of these variables, we considered an area under the curve (AUC) equivalent to 0.70 to be good, such that the percentage of monocytes was the only variable that met this criterion (0.755), with an estimated 95% confidence interval of [0.614, 0.895] and a standard error of 0.072 (see Fig. 2). The best cut-off for monocyte percentage was deemed to be the nearest score to 0.80 for sensitivity and the nearest score to 0.20 for 1-specificity, such that the best threshold for maximizing the avoidance of false positive and false negative errors was a monocyte percentage of 7.35%. The Youden test revealed that with 7.35% of monocytes, the ROC curve model (AUC = 0.755) was able to predict at best 74.3% of cases of anosognosia 6–9 months after SARS-CoV-2 infection. Interestingly, neither CRP nor basophil percentage predicted anosognosia and associated cognitive impairment.

### 3.5 Ad hoc analyses

At the reviewer's request, we performed ad hoc analyses to explore differences on immunological parameters between patients who underwent mechanical ventilation in the acute phase and those who did not (Table A2). We performed nonparametric Mann-Whitney *U* tests for continuous data, and chi-square tests for binary categorical variables, with a significance level set at *p* < .05, Bonferroni-corrected. The results are available in Supplementary Information (Tables B1 and B2).

## 4 Discussion

In the present study, we found that monocyte percentage of the total number of leukocytes in the acute phase of the disease (at hospital admission) allowed us to distinguish between patients with anosognosia for memory deficits in the chronic phase (6–9 months after SARS-CoV-2 infection) and nosognosic patients (Fig. 1 and Table 2). ROC analyses revealed that SARS-CoV-2 infected patients with a mean percentage of blood circulating monocytes above 7.35% of leucocytes measured in the acute phase predicted the presence of anosognosia in the chronic phase with high sensitivity (80%) and specificity (80%) (Fig. 2).

**Table 2:** Immunological measures (white blood cell count) for patients with COVID-19 on admission to hospital according to presence/absence of anosognosia 6–9 months post-infection.

White blood cell differential count on Day 1 of hospitalization	Anosognosic patients Mean (±*SD*)	Nosognosic patients Mean (±*SD*)	M-W or chi^2^ FDR-corrected*
Leucocytes (G/l)	5.46 (±1.98)	7.31 (±2.88)	**.020**
Lymphocytes (G/l)	0.87 (±0.37)	1.13 (±0.98)	.731
Neutrophils (G/l)	3.91 (±1.56)	5.77 (±2.71)	**.019**
Eosinophils (G/l)	0.01 (±0.04)	0.02 (±0.04)	.195
Basophils (G/l)	0.01 (±0.01)	0.02 (±0.02)	.868
Monocytes (G/l)	0.44 (±0.18)	0.37 (±0.23)	.510
Lymphocytes %	17.84 (±8.68)	16.38 (±10.82)	.481
Neutrophils %	71.16 (±11.93)	75.84 (±12.38)	.188
Eosinophils %	0.19 (±0.62)	0.27 (±0.48)	.220
Basophils %	0.20 (±0.17)	0.20 (±0.24)	.622
Monocytes %	8.29 (±2.71)	5.56 (±3.59)	.004*
Lymphocytes (below/normal/above threshold)	7/9/0	20/19/0	.612
Neutrophils (normal/above threshold)	14/2	23/16	**.041**
Eosinophils (normal/above threshold)	15/0	39/0	1
Basophils (below/above threshold)	15/0	39/0	1
Monocytes (below/above threshold)	08/07	33/6	**.016**
Lymphocyte/Monocyte ratio	2.11 (±1.03)	4.37 (±7.55)	**.018**
Lymphocyte/Neutrophil ratio	5.55 (±4.37)	10.68 (±15.74)	.359
Neutrophil/Monocyte ratio	10.62 (±6.89)	28.12 (±41.65)	**.009**
CRP	73.42 (±48.34)	99.79 (±95.37)	.678

*Note.* M-W: Mann-Whitney *U* test; FDR: false discovery rate. Immunological parameters were measured in two different units: giga per liter (G/l) and percentage of blood serum. Calculating the ratio between two immunological parameters allowed us to establish the ratio of overactivation of one parameter to that of another.

The observation that patients who exhibited anosognosia for memory functions 6–9 months after infection had a different immunological profile at the time of hospitalization supports the hypothesis of indirect damage to the central nervous system mediated by immune phenomena (Meidaninikjeh et al., 2021), leading to the development of long-term cognitive deficits (Sun et al., 2021; Visvabharathy et al., 2021). SARS-CoV-2 infection may have induced a different immunological response balance in the anosognosic patients.

Our study suggests that post-COVID-19 anosognosia may be the result of an immune imbalance in the acute phase of SARS-CoV-2 infection, particularly in terms of leukocyte distribution. In addition to the monocyte percentage, we found that both the neutrophil/monocyte ratio and the neutrophil count (G/l) tended to be lower in the acute phase of SARS-CoV-2 infection in patients who went on to develop chronic anosognosia than in nosognosic patients. Changes in blood neutrophil levels in association with neurocognitive semiology are currently being discussed in the context of neurodegenerative diseases (e.g., Alzheimer's) (Soehnlein et al., 2017) and, more recently, SARS-CoV-2 (Sarubbo et al., 2022; García-Grimshaw et al., 2022). The pro-NETotic effect of neutrophils is thought to generate an innate immune response capable of containing different infectious agents such as SARS-CoV-2 (Zuo et al., 2020). Compared with nosognosic patients, anosognosic patients had fewer neutrophils (G/l) and a lower neutrophil/monocyte ratio in the acute phase of SARS-CoV-2 infection. Different immunological mechanisms, both innate and adaptive, may be involved in the fight against viral agents like SARS-CoV-2. Regarding innate immunity, the monocyte/macrophage lineage is more likely to be involved among patients who develop anosognosia, whereas those who remain nosognosic exhibit a preponderantly neutrophilic response. We can therefore hypothesize that different brain networks and cognitive processes have different susceptibilities, depending on the type of systemic inflammatory mechanism generated in a parainfectious context.

Congruent with our observations, studies focusing on the immunological phenomena induced by SARS-CoV-2 in the acute phase have highlighted distinct immune cascades related to premorbid factors intrinsic to individual patients (Brodin, 2021). Taken together, these findings highlighting distinct immunophysiological combinations may help to explain the different trajectories observed in post-COVID-19 condition. An interesting and promising element in the understanding of this pathology is the hyperinflammation that results from excessive production of pro-inflammatory factors (Widjaja et al., 2021). Thus, cellular immunity and the production of inflammatory cytokines appear to persist in the subacute period (3 months post-infection), while inflammatory phenomena and cellular responses seem to persist 6 months post-infection. Conversely, the mechanisms of humoral immunity seem to decrease over time (Bilich et al., 2021). In line with these inflammatory hypotheses, we found a tendency for CRP and basophils to differ between patients who ended up in intensive care and those who remained in intermediate care in the acute phase, but these did not predict long-term cognitive impairments such as anosognosia or allow us to or allow us to distinguish between patients with or without these impairments. In relation to our findings, Rhally et al. (2021) showed that increased CRP is associated with vascular inflammation and altered microstructural changes in white matter. Therefore, the hypothesis of cognitive effects originating from systemic inflammation measurable using different inflammatory markers remains a central line of research in infectious contexts such as SARS-CoV-2. This interesting variation illustrates the observation that the severity of acute respiratory impairment is not a good predictor of long-term post-COVID-19 condition, at least not its cognitive aspects (Crook et al., 2021).

We therefore showed here that specific acute immunological parameters can be used to understand long-term cognitive phenomena, in particular leukocytes variations marked by the percentage of monocytes as a predictor of anosognosia. Abnormally high monocyte counts have already been observed in SARS-CoV-2 (Visvabharathy et al., 2021; Bedin et al., 2021), and have been associated with more severe disease outcomes (e.g., inflammatory amplification, impaired type I IFN production), but to our knowledge, they have never been associated with the development of cognitive impairment. Interestingly, previous studies in other pathologies have highlighted relationships between monocytic processes and cognition (Garré and Yang, 2018; Di Filippo et al., 2018; Nissen et al., 2021). Studies in HIV have highlighted relationships between increased CD14 and poorer cognitive performance, as reflected in a composite score on learning, memory, mental flexibility, verbal fluency and praxis tasks (Muñoz-Nevárez et al., 2020). Studies in multiple sclerosis (Di Filippo et al., 2018) and other neurodegenerative diseases (e.g., Alzheimer's disease or Parkinson's disease) have also shown an association between the overexpression of pro-inflammatory monocytes and decreased global cognitive performance (Garré and Yang, 2018; Nissen et al., 2021). Interestingly, encephalopathy has been observed in the acute phase of SARS-CoV-2 infection (Uginet et al., 2021), and the persistence of cognitive deficits could partly be explained by this acute-phase episode, bearing in mind that some patients may not have had a specific diagnosis of SARS-CoV-2 encephalopathy. Previous research (Andonegui et al., 2018; Michels et al., 2015; Li et al., 2020) has revealed immunopathological mechanisms roughly similar to the SARS-CoV-2 pattern in pneumonia-induced SAE. One of the key pathophysiological hypotheses concerning SAE is that the pro-inflammatory expression of monocytes can engender cognitive impairment in the long term (Andonegui et al., 2018; Chung et al., 2020; Michels et al., 2015; Trzeciak et al., 2020). Potentially relevant to COVID-19, recent studies have shown that early intervention to limit pro-inflammatory monocyte proliferation in SAE can modulate long-term cognitive deficits (Andonegui et al., 2018; Michels et al., 2015). SARS-CoV-2-related immune responses (Bedin et al., 2021) and the resulting cognitive deficits may therefore lie on the same continuum as the immune responses seen in pneumonia-induced SAE (Andonegui et al., 2018). Furthermore, it has been shown that monocytes are involved in brain damage following viral infections such as picornavirus and SARS-CoV-2 (Howe et al., 2012; Boucas et al., 2022). In particular, monocytic reactions in response to these different viral infections appear to damage hippocampal regions (Boucas et al., 2022), known to be the neuroanatomical basis of cognitive capacities in verbal episodic memory (Zammit et al., 2017).

Social lockdown measures may well have had repercussions for the immune system, and several empirical studies have indeed already highlighted the impact of lockdown on metabolism and immune processes (Filgueira et al., 2021). Physical activity may therefore be important to limit negative psychological and cognitive effects (Woods et al., 2020), as well as to improve immune defences (Filgueira et al., 2021). Interestingly, Guedj et al. (2022) showed that lockdown and social isolation during the pandemic had repercussions on the metabolic activity of sensorimotor and emotional brain areas, but these metabolic alterations were transient, and had partially disappeared 55 days after the end of lockdown. Of note, no full lockdowns were imposed in Switzerland: specific restrictions were imposed, but people were free to go outside.

One of the aims of current research is to achieve a better understanding of post-COVID-19 condition. We provide novel support for the hypothesis that immunological variations in the acute phase have repercussions on cognition 6–9 months later. Three main conclusions relating to post-COVID-19 condition can be drawn from our results. First, different immune variations may generate different cognitive deficits, as we showed that monocyte percentage predicts anosognosia for memory deficits, but other leukocyte parameters (e.g. neutrophil count) may have effects on other cognitive processes (Sarubbo et al., 2022; García-Grimshaw et al., 2022). Second, at a time when an increasing number of people are living with post-COVID-19 condition (Gorna et al., 2021; Sudre et al., 2021), our results suggest that it would be possible to plan neuropsychological follow-up on the basis of acute leukocyte markers as soon as patients with COVID-19 are hospitalized. The provision of this follow-up would allow hospital structures to be better organized, with more specialist support being given to patients. Third, recent studies have indicated that SARS-CoV-2 infection may be a trigger for neurodegenerative pathologies (e.g., Alzheimer's disease), as well as a catalyst for neurodegenerative processes, just like other HCoVs (Meessen-Pinard et al., 2017; Dolatshahi et al., 2021). In line with Voruz et al. (2022a), we argue that the cognitive profiles of our anosognosic patients were very similar to those observed in Alzheimer's disease (Starkstein, 2014), suggesting the development of a cognitive precursor to a neurodegenerative pathology. The mechanisms that trigger neurodegenerative pathologies are not yet well understood. However, one hypothesis suggests that neurodegenerative pathologies can be triggered by infiltration of the nervous system by microglia (Baufeld et al., 2018) following inflammatory responses. This hypothesis could be applied to SARS-CoV-2 (Sun et al., 2021; Li et al., 2021). Thus, the high level of monocytes observed both in our study and in previous studies of patients with SARS-CoV-2 (Bedin et al., 2021) could be a risk factor for the development of neurodegenerative processes. Further studies are needed to look for markers of neurodegenerative pathologies in patients with COVID-19.

Our study had four limitations. First, although no other study has yet attempted to link cognitive and immunological variables in SARS-CoV-2, our sample of 61 patients could be considered small. However, as we describe in Section 2 (Method), our power analysis based on two conditions (i.e., sepsis and HIV) where authors have sought to establish a link between cognition and immunology revealed that we had sufficient participants in this cohort of patients with COVID-19. Second, our measure of anosognosia could be subject to debate (Starkstein, 2014). Anosognosia is difficult to measure, despite important advances in its understanding in mild cognitive impairment and Alzheimer's disease (Starkstein, 2014; Tondelli et al., 2018-). It can be measured (i) by the clinician, using a clinical assessment, (ii) as the discrepancy between the patient's subjective complaints and objective neuropsychological scores, or (iii) as the difference between the patient's complaints and the caregiver's assessment in terms of activities of daily living (Starkstein, 2014). In our study, we used two measures of anosognosia (clinical assessment and calculation of self-appraisal discrepancy score). Third, although we included patients who were free of relevant medical history before the infection, and we retrospectively extracted their physiological variables on admission to hospital, to avoid the effect of any treatment for COVID-19, their immunological characteristics may have been modulated by treatments taken beforehand. Fourth, all the patients included in the study were infected by the original strain of SARS-CoV-2, and subsequent variants of the virus could have different cognitive and immunological implications.

## 5 Conclusion

We conducted the first retrospective analysis to establish a relationship between immunological responses in the acute phase of SARS-CoV-2 infection and long-term cognitive deficits 6–9 months after infection, including anosognosia for memory impairment. Our results could be of great importance for the future management of patients, as well as for understanding the emergence of post-COVID-19 condition. A high percentage of blood circulating monocytes could be a predictor of neurological post-COVID-19 condition, unlike disease severity in the acute phase, which is not predictive of long-term cognitive effects. Limiting monocyte proliferation could be a means of limiting long-term cognitive impairment following SARS-CoV-2 infection, but further research is required.

## Declaration of competing interest

The authors report no conflicts of interest.

## Data Availability

Nonsensitive COVID-COG data will be made available at the end of the project on a dedicated open-access platform.
