# Supplementary material for: Monocytosis in the acute phase of SARS-CoV-2 infection predicts the presence of anosognosia for cognitive deficits in the chronic phase
Source: Brain Behav Immun Health. 2022 Sep 16;26:100511. doi: 10.1016/j.bbih.2022.100511 (PMC9477785; doi:10.1016/j.bbih.2022.100511)
Supplement: Multimedia component 1 [file mmc1.docx]

**Supplementary Information A**

**Table A1) Numbers of anosognosic and nosognosic patients for whom immunological data were available**

| Immunology on Day 1 of hospitalization | *n* anosognosic patients | *n* nosognosic patients |
| --- | --- | --- |
| **Leucocytes (G/l)** | 20 | 41 |
| **Lymphocytes (G/l)** | 18 | 39 |
| **Neutrophils (G/l)** | 16 | 39 |
| **Eosinophils (G/l)** | 15 | 39 |
| **Basophils (G/l)** | 15 | 39 |
| **Monocytes (G/l)** | 15 | 39 |
| **Lymphocytes %** | 16 | 39 |
| **Neutrophils %** | 16 | 39 |
| **Eosinophils %** | 15 | 39 |
| **Basophils %** | 15 | 39 |
| **Monocytes %** | 15 | 39 |
| **Lymphocytes (below/above threshold)** | 16 | 39 |
| **Neutrophils (below/above threshold)** | 16 | 39 |
| **Eosinophils (below/above threshold)** | 15 | 39 |
| **Basophils (below/above threshold)** | 15 | 39 |
| **Monocytes (below/above threshold)** | 15 | 39 |
| **Lymphocyte/Monocyte ratio** | 15 | 38 |
| **Lymphocyte/Neutrophil ratio** | 16 | 39 |
| **Neutrophil/Monocyte ratio** | 15 | 38 |
| **Thrombocytes (G/l)** | 20 | 41 |
| **CRP** | 20 | 41 |
| **Sodium (mmol/l)** | 20 | 41 |
| **Potassium (mmol/l)** | 20 | 41 |
| **Creatinine (micromole/l)** | 20 | 41 |
| **Erythrocytes (T/l)** | 20 | 41 |
| **Hemoglobin (G/l)** | 20 | 41 |

*Note.* Immunological parameters were measured in two different units: Giga per liter (G/l) and percentage of blood serum. Calculating the ratio between two immunological parameters allowed us to know the ratio of overactivation of one parameter to that of another. Millimole (mmol) is the unit of measurement of matter at 10^-3^ or 10^-6^, and (T/l) is the SI unit for measuring red blood cells.

**Table A2) Sociodemographic variables according to severity of respiratory symptoms (hospitalized patients without mechanical ventilation vs. mechanical ventilation in ICU)**

|  | Hospitalized patients without mechanical ventilation | ICU patients with mechanical ventilation | FDR-corrected *p*. value *  (M-W or chi^2^) |
| --- | --- | --- | --- |
|  | *n =* 36 | *n =* 25 |  |
| Mean length of hospitalization in days | 12.125 | 37.25 | **.000*** |
| History of respiratory problems | 3/36 | 6/25 | .092 |
| Cardiovascular history | 6/36 | 6/25 | .48 |
| Neurological history | 0/36 | 0/25 | 1 |
| Psychiatric history | 1/36 | 1/25 | 0.79 |
| History of developmental disorder | 0/36 | 0/25 | 1 |
| Sleep apnea syndrome) | 1/36 | 8/25 | **.007*** |
| Chronic renal failure | 0/36 | 2/25 | .087 |
| Immunosuppression | 0/36 | 0/25 | 1 |
| Pregnant | 0/36 | 0/25 | 1 |
| Left lateralization | 3/36 | 1/25 | .50 |
| Mean age in years | 56.1 | 61.8 | .078 |
| Education level (1/2/3) | 2/9/25 | 1/11/13 | .214 |
| Sex (female) | 14/36 | 5/25 | .120 |

*Note.* Education level: 1 = compulsory schooling, 2 = post-compulsory schooling, and 3 = university degree or equivalent. ICU: intensive care unit; M-W: Mann‑Whitney *U* test. Results in bold and marked * were significant after FDR correction.

**Supplementary Information B**

**Table B1) Numbers of patients in ICU with mechanical ventilation and hospitalized patients without mechanical ventilation**

| Immunology on Day 1 of hospitalization | *n* hospitalized patients without mechanical ventilation | *n* ICU patients with mechanical ventilation |
| --- | --- | --- |
| **Leucocytes (G/l)** | 36 | 25 |
| **Lymphocytes (G/l)** | 32 | 23 |
| **Neutrophils (G/l)** | 32 | 22 |
| **Eosinophils (G/l)** | 33 | 23 |
| **Basophils (G/l)** | 32 | 23 |
| **Monocytes (G/l)** | 33 | 23 |
| **Lymphocytes %** | 32 | 23 |
| **Neutrophils %** | 32 | 23 |
| **Eosinophils %** | 31 | 23 |
| **Basophils %** | 31 | 23 |
| **Monocytes %** | 31 | 23 |
| **Lymphocyte/Monocyte ratio** | 30 | 23 |
| **Lymphocyte/Neutrophil ratio** | 32 | 23 |
| **Neutrophil/Monocyte ratio** | 30 | 23 |
| **CRP** | 36 | 25 |

**Table B2) Immunological measures (white blood cell count) of patients with COVID-19 on admission to hospital according to whether they were hospitalized in ICU with mechanical ventilation or hospitalized without mechanical ventilation**

| White blood cell differential count on Day 1 of hospitalization | ICU patients with mechanical ventilation  Mean (± *SD*) | Hospitalized patients without mechanical ventilation  Mean (± *SD*) | Bonferroni-corrected *p* value  (M-W or chi^2^) |
| --- | --- | --- | --- |
| **Leucocytes (G/l)** | 6.11 (± 1.95) | 7.21 (± 3.09) | .76 |
| **Lymphocytes (G/l)** | 0.82 (± 0.44) | 1.23 (± 1.02) | .14 |
| **Neutrophils (G/l)** | 4.75 (± 1.96) | 5.45 (±2.68) | .44 |
| **Eosinophils (G/l)** | 0.20 (± 0.04) | 0.10 (±0.20) | .68 |
| **Basophils (G/l)** | 0.016 (± 0.018) | 0.009 (±.009) | .069 |
| **Monocytes (G/l)** | 0.36 (± 0.17) | 0.41 (±0.24) | .87 |
| **Lymphocytes %** | 15.54 (± 9.71) | 17.45 (± 10.09) | .52 |
| **Neutrophils %** | 75.74 (± 11.83) | 73.96 (± 10.97) | .72 |
| **Eosinophils %** | 0.33 (± 0.67) | 0.13 (± 0.22) | .63 |
| **Basophils %** | 0.28 (± 0.28) | 0.14 (± 0.15) | **.022** |
| **Monocytes %** | 6.58 (± 3.49) | 6.34 (± 3.60) | .71 |
| **Lymphocyte/Monocyte ratio** | 2.53 (± 1.18) | 4.61 (± 8.50) | .50 |
| **Lymphocyte/Neutrophil ratio** | 7.66 (± 5.59) | 9.43 (± 16.19) | .59 |
| **Neutrophil/Monocyte ratio** | 17.43 (± 13.48) | 27.73 (± 46.55) | .96 |
| **CRP** | 113,22 (± 84.67) | 77.69 (± 83.73) | **.026** |

*Note:* Results in bold were significant before Bonferroni correction. M-W: Mann‑Whitney *U* test.
